# Supplementary material for: Modeling glioblastoma heterogeneity as a dynamic network of cell states
Source: Mol Syst Biol. 2021 Sep 16;17(9):e10105. doi: 10.15252/msb.202010105 (PMC8444284; doi:10.15252/msb.202010105)
Supplement: Supplementary file 6 — Source Data for Figure 5 [file MSB-17-e10105-s004.zip › Figure5A_sourcedata/GSEA_3071/nosigdb_stateV.GseaPreranked.1622795159954/NB_KI_CLUSTER11.html]

Details for gene set NB\_KI\_CLUSTER11[GSEA]

|  || Dataset | state43071 |
| Phenotype | NoPhenotypeAvailable |
| Upregulated in class | na\_neg |
| GeneSet | NB\_KI\_CLUSTER11 |
| Enrichment Score (ES) | -0.7005193 |
| Normalized Enrichment Score (NES) | -3.1362152 |
| Nominal p-value | 0.0 |
| FDR q-value | 0.0 |
| FWER p-Value | 0.0 |
Table: GSEA Results Summary

  

Fig 1: Enrichment plot: NB\_KI\_CLUSTER11      
 Profile of the Running ES Score & Positions of GeneSet Members on the Rank Ordered List

  

| PROBE | GENE SYMBOL | GENE\_TITLE | RANK IN GENE LIST | RANK METRIC SCORE | RUNNING ES | CORE ENRICHMENT || 1 | GYPC |  |  | 97 | 1.375 | 0.0188 | No |
| 2 | LITAF |  |  | 412 | 0.939 | -0.0155 | No |
| 3 | TGIF1 |  |  | 840 | 0.683 | -0.0782 | No |
| 4 | IER2 |  |  | 1742 | 0.428 | -0.2380 | No |
| 5 | ANXA1 |  |  | 2165 | 0.366 | -0.3084 | No |
| 6 | EEF1D |  |  | 2307 | 0.349 | -0.3257 | No |
| 7 | CCND2 |  |  | 2876 | 0.282 | -0.4261 | No |
| 8 | IFITM2 |  |  | 3585 | -0.312 | -0.5523 | No |
| 9 | AKAP13 |  |  | 4289 | -0.470 | -0.6733 | No |
| 10 | B2M |  |  | 4341 | -0.485 | -0.6699 | No |
| 11 | PSMB9 |  |  | 4503 | -0.543 | -0.6858 | Yes |
| 12 | CASP4 |  |  | 4551 | -0.565 | -0.6794 | Yes |
| 13 | ISG15 |  |  | 4586 | -0.579 | -0.6702 | Yes |
| 14 | AC020916.1 |  |  | 4620 | -0.604 | -0.6601 | Yes |
| 15 | MSN |  |  | 4698 | -0.644 | -0.6573 | Yes |
| 16 | PNRC1 |  |  | 4813 | -0.726 | -0.6593 | Yes |
| 17 | ARPC1B |  |  | 4818 | -0.730 | -0.6403 | Yes |
| 18 | CLIC1 |  |  | 4899 | -0.809 | -0.6336 | Yes |
| 19 | SLC2A3 |  |  | 4942 | -0.859 | -0.6183 | Yes |
| 20 | TGFB1 |  |  | 4944 | -0.861 | -0.5952 | Yes |
| 21 | FLNA |  |  | 4948 | -0.862 | -0.5724 | Yes |
| 22 | PPP1R15A |  |  | 4966 | -0.882 | -0.5517 | Yes |
| 23 | TSPO |  |  | 4970 | -0.885 | -0.5283 | Yes |
| 24 | IL32 |  |  | 5006 | -0.946 | -0.5093 | Yes |
| 25 | S100A10 |  |  | 5011 | -0.954 | -0.4842 | Yes |
| 26 | SH3BGRL3 |  |  | 5063 | -1.064 | -0.4651 | Yes |
| 27 | KLF2 |  |  | 5083 | -1.110 | -0.4386 | Yes |
| 28 | EMP3 |  |  | 5084 | -1.113 | -0.4085 | Yes |
| 29 | IRF1 |  |  | 5100 | -1.155 | -0.3800 | Yes |
| 30 | GLIPR1 |  |  | 5109 | -1.177 | -0.3497 | Yes |
| 31 | SP100 |  |  | 5126 | -1.230 | -0.3194 | Yes |
| 32 | S100A6 |  |  | 5183 | -1.473 | -0.2901 | Yes |
| 33 | PLAUR |  |  | 5185 | -1.479 | -0.2502 | Yes |
| 34 | S100A11 |  |  | 5196 | -1.536 | -0.2105 | Yes |
| 35 | THBS1 |  |  | 5197 | -1.538 | -0.1688 | Yes |
| 36 | RARRES3 |  |  | 5206 | -1.582 | -0.1275 | Yes |
| 37 | CIB1 |  |  | 5220 | -1.664 | -0.0848 | Yes |
| 38 | S100A4 |  |  | 5238 | -1.817 | -0.0388 | Yes |
| 39 | MYL12A |  |  | 5239 | -1.826 | 0.0107 | Yes |
Table: GSEA details [plain text format]

  

Fig 2: NB\_KI\_CLUSTER11: Random ES distribution      
 Gene set null distribution of ES for **NB\_KI\_CLUSTER11**
